# Correlation of histopathological diagnosis with habits and clinical findings in oral submucous fibrosis

**DOI:** 10.1186/1758-3284-1-10

**Published:** 2009-05-02

**Authors:** Shruti Pandya, Ajay Kumar Chaudhary, Mamta Singh, Mangal Singh, Ravi Mehrotra

**Affiliations:** 1 Departments of Pathology, Moti Lal Nehru Medical College, Allahabad, India; 2 Otorhinolayngology, Moti Lal Nehru Medical College, Allahabad, India; 3 Centre for Biotechnology, University of Allahabad, India

## Abstract

**Background:**

Oral submucous fibrosis is a common oral health problem in India. This study was conducted to correlate the histopathological diagnosis with habits and clinical findings in patients suffering from oral submucous fibrosis (OSF).

**Methods:**

Patients suffering from oral submucous fibrosis from the Departments of Otorhinolayngology and Pathology, Moti Lal Nehru Medical College, Allahabad, India were studied from 2004–2008. Detailed information was gathered in a pretested proforma. Emphasis was given to the various addictions, clinical findings and histological examination was done.

**Results:**

Two hundred and thirty nine patients were studied, yielding a male to female ratio of 6.8:1. Maximum patients were in the 21–30 years age group with a marked male predominance. Of these, 197 (82.4%) patients chewed areca nut/dohra, 14 (5.8%) were smokers and 2 (0.8%) patients were habituated to alcohol. 89(37.2%) patients reported difficulty in opening of the mouth (trismus). 51 (57.4%) patients were found to have stage II (2–3 cm) trismus while rest had stage I and III. The buccal mucosa was found to be the most commonly involved site. On the basis of histopathological examination, 52(21.7%) were classified as OSF grade I, 75(31.3%) patients as grade II and 112(46.8%) had grade III disease.

**Conclusion:**

The widespread habit of chewing dohra/paan masala is a major risk factor of OSF, especially in the younger age group. In this study, an increase in histopathological grading was found with severity and duration of addiction habit. However no significant correlation was found between clinical staging and histopathological grading.

## Background

Oral submucous fibrosis (OSF) is a chronic and potentially malignant condition of the oral cavity. It is characterized by a juxtraepithelial inflammatory reaction followed by fibroelastic changes in the lamina propia and associated epithelial atrophy. The disease affects most part of the oral cavity as well as the upper third of the esophagus. [1]

The pathogenesis of OSF is not well established, but is believed to be multifactorial. The chewing of betel quid (containing areca nut, tobacco and slaked lime) has been recognized as one of the most important risk factors for OSF. [2-4] Over the years, the incidence of OSF has increased manifold in various parts of the Indian subcontinent including Allahabad. [5]

In spite of the fact that the habit of areca nut chewing with or without betel quid is rampant, the correlation between the extent and duration of addictions with clinical and histopathological grading has not been attempted so far. Thus, this study was designed investigate to these issues.

## Methods

Patients suffering from OSF from the Departments of Otorhinolayngology and Pathology, Moti Lal Nehru Medical College, Allahabad were studied from 2004–2008, after obtaining clearance from the institutional ethical committee. Detailed information of each patient was noted in a pretested proforma. Information regarding the patients' name, age, sex, occupation, background, dietary habits, dental hygiene, personal habits and present complaints was gathered. Emphasis was given to addictions like areca nut, tobacco and alcohol. Detailed clinical examination of each patient was done to assess the site, size and type of lesion. Trismus was classified as stage I (> 3 cm), stage II (2–3 cm) and stage III (<2 cm). For confirmation of the clinical diagnosis, histopathological examination was carried out. The biopsy tissue was processed by paraffin embedding and 2–3 micrometer thick sections were cut and stained by Haematoxylin and eosin (H and E). Histopathological examination was done and results were recorded according to the traditional grading by Pindborg and Sirsat. [6] who had described four consecutive stages (Table 1)

**Table 1:** Histopathological classification of OSF

Very early stage (Grade I):	Early stage (Grade II):	Moderately advanced stage (Grade III):	Advanced stage (Grade IV):
❖ A finely fibrillar collagen, dispersed with marked edema. ❖ The fibroblastic response is strong. ❖ The blood vessels are sometimes normal, but more often they are dilated and congested. ❖ Inflammatory cells, mainly polymorphonuclear leukocytes with an occasional eosinophil are present.	❖ The juxta-epithelial area shows early hyalinization. ❖ Plump young fibroblasts are present in moderate numbers. ❖ The blood vessels are dilated and congested. ❖ The inflammatory cells are mostly mononuclear lymphocytes, eosinophils and an occasional plasma cell.	❖ The collagen is moderately hyalinized. ❖ The fibroblastic response is less marked, the cells present being mostly adult fibrocytes. ❖ Blood vessels are normal or constricted. ❖ The inflammatory exudates consist of lymphocytes and plasma cells, although an occasional eosinophil is seen.	❖ The collagen is completely hyalinized. ❖ The hyalinized areas are devoid of fibroblasts. ❖ Blood vessels are completely obliterated or narrowed. ❖ The inflammatory cells are lymphocytes and plasma cells.

## Results

**Figure 1:**
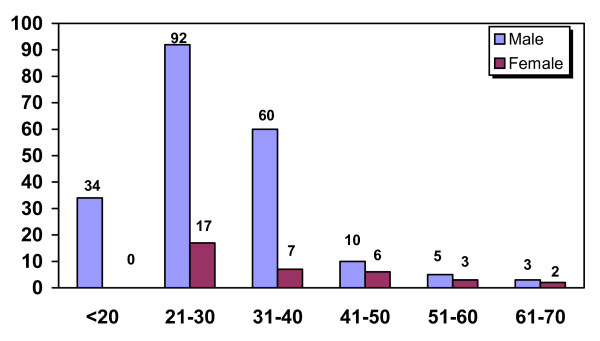
**Distribution of 239 cases of OSF according to age & sex**.

Two hundred and thirty nine OSF patients were studied, of which 204(85.4%) were males and 35(14.6%) females with a male to female ratio of 6.8:1. Maximum number of patients, 109(45.6%) were in the 21–30 years age group followed by 67 (28%) patients in the 31–40 years of age [Figure 1].

According to their personal habits, 110(46%) patients chewed areca nut/dohra, 49 (20.5%) patients consumed gutka, 38 (15.8%) patients were habituated to smoking, 11(4.6%) chewed and smoked, 7 (2.9%) patients were addicted to alcohol and chewing. 7 (2.9%) patients were addicted to alcohol and smoking and 2 patients were addicted only to alcohol [Table 2].

**Table 2:** Distribution of patients on their personal habits

Personal Habits		No. of Patients	Total
Chewing	(Areca Nut/Dohra)	110 (46%)	197(82.4%)
		
	Gutka	49 (20.5%)	
		
	Betel quid with areca nut and tobacco	38 (15.8%)	

Smoking	Bidi/Cigarettes/Pipes	14 (5.9%)	14 (5.9%)

Alcohol		2 (0.8%)	2 (0.8%)

Combination	Chewing + Smoking	11 (4.6%)	25 (10.4%)
		
	Alcohol+ Chewing	7 (2.9%)	
		
	Alcohol+ Smoking	7 (2.9%)	

None		1 (0.4%)	

Total		239	

**Figure 2:**
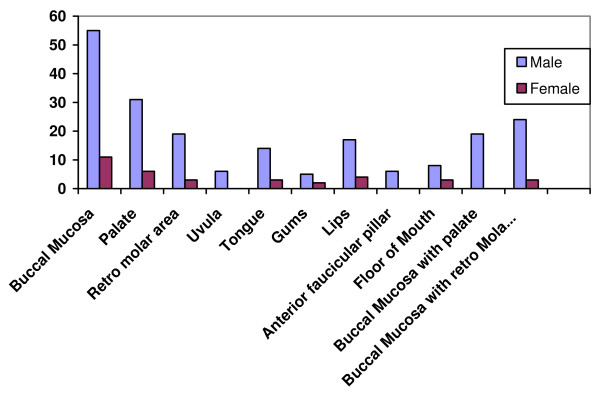
**Sites of fibrosis**.

With regard to site distribution, the buccal mucosa was the most common involved site with 66(20.8%) patients being involved. Palate was the second common site and affected 37(17.7%) patients. Both buccal mucosa and the palate were involved in 19(7.9%) patients. Buccal mucosa with the retro molar area involvement was found in 27 (3.1%) patients [Figure 2].

**Figure 3:**
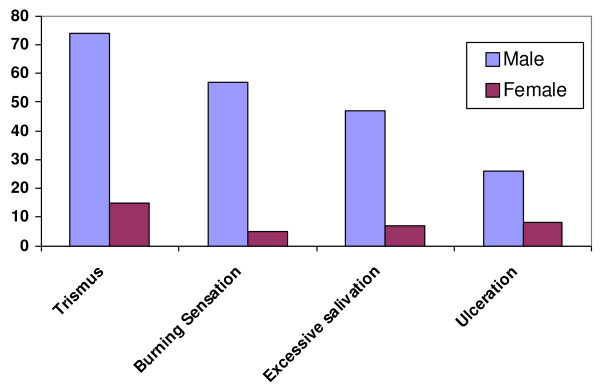
**Gender wise distribution of patients with their clinical symptoms**.

On the basis of clinical symptoms, 89(37.2%) patients reported difficulty in opening of the mouth, 62(25.9%) patients suffered from a burning sensation of the buccal cavity, 54(22.5%) patients reported excessive salivation and 34(14.2%) patients reported recurrent oral ulcerations [Figure 3].

On correlating the histopathological findings with the patients' addiction habits, 52 (21.7%) had OSF grade I, of which 20(38.5%) chewed paan masala/dohra, 10 (19.2%) took only gutka, 7(13.5%) consumed betel quid with areca nut and tobacco, 5 (11.1%) were smokers, 5 (9.6%) patients were both chewers and smokers, 3 (5.8%) were addicted to alcohol and chewing, while 2 (3.6%) were habituated to alcohol and smoking. On correlating histopathological findings with frequency and duration of addiction in OSF grade I, maximum patients were addicted for at least 3–5 years and used tobacco products 4–5 times per day.

**Figure 4:**
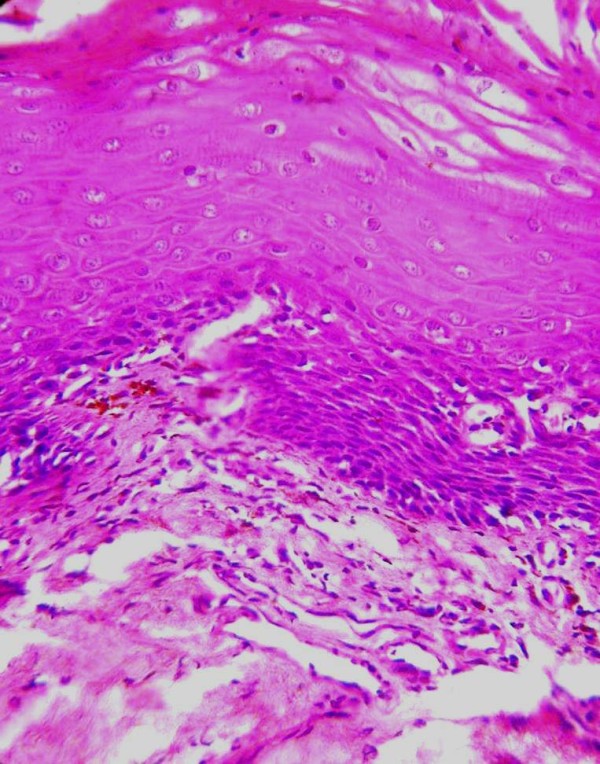
**Oral mucosa showing OSF grade II demonstrating fibroblasts in the juxta-epithelial area with dilated blood vessels (H and E ×100)**.

In OSF grade II category, out of 75(31.3%) patients, 30 (40%) chewed paan masala/dohra, 16(21.3%) were habituated to gutka, 11 (14.7%) took betel quid along with areca nut and tobacco, 6(8%) smoked bidi/cigarettes [Figure 4]. Four (5.3%) patients chewed and smoked tobacco, 3(4%) were addicted to chewing and alcohol and another 3 (4%) were addicted to alcohol and smoking. One patient was addicted to alcohol and 1 patient did not have any habit. In this group, maximum patients were addicted for 7–10 years and daily consumed the substances 4–8 times per day.

**Figure 5:**
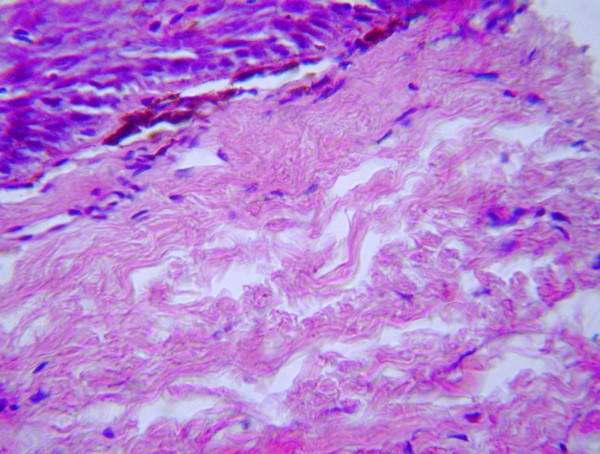
**Oral Mucosa showing OSF grade III showing thinning of the epidermis, hyalinized collagen and lymphocytic infiltration**. (H and E × 400).

One hundred and twelve (46.8%) patients had histopathological grade III disease and of these 60 (53.5%) chewed paan masala/dohra, 23 (20.4%) were habituated to gutka, 20 (18%) consumed betel quid with areca nut and tobacco, 3(2.7%) were smokers, 2 (1.8%) both chewed and smoked, 2 (1.8%) were addicted to alcohol and smoking, 1(0.9%) consumed alcohol and chewed tobacco and 1(0.9%) was addicted to alcohol. Majority of patients of this group consumed tobacco products for 8–10 years or more with a frequency of 6–10 times per day [Table 3, Figure 5].

**Table 3:** Distribution of patients with their Histopathological findings and habits

Histological Findings	No of patients N = 239	Habits	No of Patients	Male N = 204	Female N = 35	Frequency/Day	Duration (Years)
OSMF I	52 (21.8%)	Chewing (Areca Nut/Dohra)	20 (38.5%)	17 (85%)	3 (15%)	3–4	3–4 yrs
		
		Gutka	10 (19.2%)	9 (90%)	1 (10%)	2–3	2–4 yrs
		
		Betel quid with areca nut and tobacco	7 (13.5%)	4 (57.2%)	3 (42.8%)	2–4	4–5 yrs
		
		Smoking (Bidi/Cigarettes/Pipes	5 (11.1%)	5 (100%)	0 (0%)	5–10	>5 yrs
		
		Alcohol	0 (0%)	0 (0%)	0 (0%)	0	0
		
		Chewing + Smoking	5 (9.6%)	4 (80%)	1 (20%)	2–5/	3–4 yrs
		
		Alcohol+ Chewing	3 (5.8%)	3 (100%)	0 (0%)	1–3	2–3 yrs
		
		Alcohol+ Smoking	2 (3.6%)	2 (100%)	0 (0%)	2–5	4–5 yrs

OSMF II	75 (31. 4%)	Chewing (Areca Nut/Dohra)	30 (40%)	24 (80%)	6 (20%)	7–10	8–10 yrs
		
		Gutka	16 (21.3%)	11(68.8%)	5 (31.2%)	5–6	7–10 yrs
		
		Betel quid with areca nut and tobacco	11 (14.7%)	7 (63.7%)	4 (36.3%)	6–9	6–7 yrs
		
		Smoking (Bidi/Cigarettes/Pipes)	6 (8%)	6 (100%)	0 (0%)	8–10	7–9 yrs
		
		Alcohol	1 (1.3%)	1 (100%)	0 (0%)	2–3	6–7
		
		Chewing + Smoking	4 (5.3%)	3 (75%)	1 (25%)	4–8	6–8 yrs
		
		Alcohol+ Chewing	3 (4%)	3 (100%)	0 (0%)	3–8	7–10 yrs
		
		Alcohol+ Smoking	3 (4%)	3 (100%)	0 (0%)	4–7	8–10 yrs
		
		None	1 (1.4%)	1 (100%)	0 (0%)		

OSMF III	112 (46.8%)	Chewing(Areca Nut/Dohra)	60 (53.5%)	54 (90%)	6 (10%)	10–15	10–17 yrs
		Gutka	23 (20.4%)	21(91.3%)	2(8.7%)	8–10	7–10 yrs
		
		Betel quid with areca nut and tobacco	20 (18%)	17 (85%)	3 (15%)	7–9	7–9 yrs
		
		Smoking (Bidi/Cigarettes/Pipes)	3(2.7%)	3 (100%)	0 (0%)	8–10	9–10 yrs
		
		Alcohol	1(0.9%)	1 (100%)	0 (0%)	4–5	7–10
		
		Chewing + Smoking	2(1.8%)	2 (100%)	0 (0%)	6–9	8–10 yrs
		
		Alcohol+ Chewing	1(0.9%)	1 (100%)	0 (0%)	4–8	7– 9 yrs
		
		Alcohol+ Smoking	2(1.8%)	2 (100%)	0 (0%)	6–9	8–10 yrs

On correlating clinical grading of trismus and histopathological grading, patients who had clinical stage I trismus 3(16.6%) patients had grade I, 5(17.2%) had grade II and 8 (19%) had grade III. In stage II trismus group, 11(61.1%) patients had grade I and 17(58.6%) had grade II and 23(54.7%) had grade III. While in stage III trismus, 4 (22.2%) patients had grade I, 7 (24.1%) had grade II and 11(26.1%) had grade III. [Table 4]

**Table 4:** Distribution of patients according to staging of trismus and histopathological grading

Stage of Trismus	Grading			Total
	**Grade I**	**Grade II**	**Grade III**	

Stage I	3 (16.6%)	5 (17.2%)	8 (19%)	16 (17.9%)

Stage II	11 (61.1%)	17 (58.6%)	23 (54.7%)	51(57.3%)

Stage III	4 (22.2%)	7 (24.1%)	11 (26.1%)	22 (24.7%)

Total	18 (20.2%)	29 (32.5%)	42 (47.1%)	89

## Discussion

OSF is a potentially malignant disease of oral cavity and is most commonly found in Asian countries. Reichart et al suggested that as a result of transmigration of populations, an increasing number of OSF cases are being found in other countries. [7] It constitutes one of the major oral health problems in countries like India. In this study, 239 OSF patients were studied over a 4-year period. Majority of the patients were in the 21–30 years of age group with a male to female ratio 6.8:1. Kumar et al found similar results from Chennai. [8] Hazarey et al from Nagpur also reported that most of their patients were in the younger age group (< 30 years) with a similar male to female ratio of 5:1. [9] However, Zhang et al from China suggested that the prevalence of betel quid chewing is highest in the Hunan and Hainan provinces (64.5% to 82.7%) with signs of OSF in 0.9% to 4.7% of the population and the 30 to 49 years age group being the most commonly affected [10]

**Figure 6:**
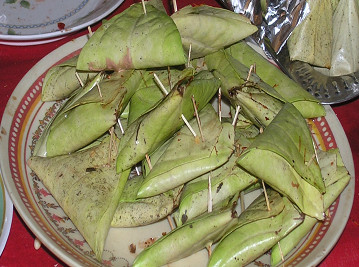
**Paan quids being served at a wedding reception**.

Areca nut, incriminated in the causation of OSF is often wrapped in the leaf of a tropical creeper, *Piper betle *L. commonly known as the betel leaf or paan [Figure 6]. The usage of paan is widespread in the Indian subcontinent, mostly in the Hindi speaking heartland of North and Central India.

In the Allahabad region, consumption of a unique preparation called dohra is widespread. [11] It is popular in the district as well as neighbouring regions of Jaunpur and Pratapgarh. It is a mixture of tobacco, slaked lime, areca nut and other ingredients like catechu (*katha*), peppermint and cardomom (illayachi)etc*.*It is a wet preparation and marketed without any brand name. About 200 mg product is kept in plastic bag and a rubber band is applied. One packet is sold for as less as one rupee (approx two US cents). Users consume tobacco (Surti/Zarda) with dohra according to their level of addiction. In this study, 110 (46%) patients, chewed paan masala/dohra. On the other hand, Kumar et al reported from Chennai that 81% of their patient's had the habit of chewing raw areca nut/commercial areca nut/paan masala. [8] Hazarey et al reported in their study from Nagpur in Western India that areca nut in its pure form was more commonly consumed by women while Khara/Mawa, the common name of gutka (combination of areca nut, paan masala and tobacco) in that region, was usually consumed by men. [9] Babu et al reported that habitual chewing of pan masala/gutkha is associated with earlier presentation of oral submucous fibrosis than betel quid use. [12] Thomas et al from South India suggested tobacco chewing was the most important risk factor for multiple oral premalignant lesions and may be a major etiological factor for cancers on the oral epithelium in the Indian population. [13]

In this study, 38 (15.8%) patients were addicted to betel quid with areca nut and tobacco.14 (5.9%) males were addicted to smoking alone. Only 2 (0.8%) males were habituated to alcohol, but no consistent correlation was found between the OSF and smoking/alcohol consumption. Ho et al reported a significant contribution of smoking and alcohol consumption to the malignant transformation of OSF [14] However, combination of alcohol, chewing and smoking was comparatively more dangerous, 25 (10.4%) patients were addicted to combination of chewing, smoking and alcohol. Similarly, Auluck et al reported from immigrant population in Canada that smoking and alcohol drinking along with areca quid chewing showed a significant association with leukoplakia, OSF and verrucous lesions. [15]

Buccal mucosa was found the most commonly involved site in 66(20.8%) patients followed by palate 37(17.7%) and the retromolar area 22(14.7%). Previous reports also corroborated these findings. [9,14,15] Bhugari et al from Pakistan also reported that mucosa of the cheek (55.9%) was the most common site followed by the tongue (28.4%) [16] While Reichart and Way reported the tongue was the most common site, in their study. [17] In this series, none of the patients were reported with involvement of the larynx, pharynx or the esophagus.

Clinically, trismus (opening of the mouth cavity) is an important symptom of OSF. In this study, 89 (37.2%) patients were found to have trismus of which, 16 (17.9%) had stage I, 51(57.3%) patients had stage II trismus followed by 22 (24.7%) of stage III. Chiu et al reported the trismus was the chief complaint in 90.8% of their patients. [18] Kumar et al also reported that 75% males and 80% females with OSF patients had stage II disease and suggested that this could be due to the fact that the majority of the patients reported for treatment only after the onset of restriction in their ability to open their mouths. [8] Hazarey et al also reported that maximum patients of OSF, in their study, had stage III trismus. [9]

On the correlation of addiction habit and histopathological findings, maximum patients had histopathological grade III OSF and took tobacco products for 8–10 years or more with high frequency (7–10 times per day) followed by histopathological grade II and I. Kumar et al suggested the patients who used paan masala with a greater frequency/day developed OSF with a shorter duration of the habit. [8] Maher et al from Pakistan reported that the daily consumption rate appears to be much more significant with respect to risk than the lifelong duration of the habit. [19] Some reports suggested that both the duration and daily frequency of areca nut use increase the risk of cancer, suggesting a dose-response relationship. [20] Similarly, Shah et al reported that the total duration of the chewing habit was not significantly correlated to OSF. They hypothesized that the exposure to the total burden of various harmful substances in a given period, *i.e.*, daily consumption was more significant that the total duration of the habit. [21] No correlation was found between clinical grading and histopathological grading in this study akin to Kumar et al who did not find any correlation between clinical symptoms and degree of fibrosis. [8]

The treatment of patients with oral submucous fibrosis depends on the degree of clinical involvement. If the disease is detected at a very early stage, cessation of the habit is sufficient. Most patients with oral submucous fibrosis present with moderate-to-severe disease. Medical treatment is symptomatic and predominantly aimed at improving mouth movements. Treatment strategies include the following: Steroids, Placental extracts [22], Hyaluronidase, Pentoxifylline[23], IFN-gamma[24] and Lycopene[25].

Surgical treatment is indicated in patients with severe trismus and/or biopsy results revealing dysplastic or neoplastic changes. Surgical modalities that have been used include simple excision of the fibrous bands, Split-thickness skin grafting, Nasolabial flaps and lingual pedicle flaps. Use of a KTP-532 laser release procedure was recently found to increase mouth opening range in 9 patients over a 12-month follow-up period in one study [26].

Physical therapy using muscle-stretching exercises for the mouth may be helpful in preventing further limitation of mouth movements. This is often combined with medical and surgical therapy.

Surveillance for OSF is being carried out routinely in the department of Otorhinolaryngology out-patients department at the S.R.N. Hospital associated with the Medical School. As a small percentage of patients with OSF go on to develop malignancy, correlation of histopathological findings and clinical findings is important.

## Conclusion

This study concluded that the widespread habits of chewing dohra and paan masala are the major risk factors of OSF, especially affecting the younger generation. An increase is found in histopathological grading and addiction habit and to the best of our knowledge, this correlation has not been attempted before. However no significant correlation was found between trismus and histopathological grading.

## Competing interests

The authors declare that they have no competing interests.

## Authors' contributions

SP and AKC carried out the experimental work, analysis and drafted the manuscript. RM conceived of the study, participated in its design and coordination as well as helped to draft the manuscript. MS and Mamta Singh participated in coordination of the study and helped to draft the manuscript. All authors read and approved the final manuscript.

## References

[B1] Tilakratne WM, Klinikowski MF, Saku T, Peters TJ, Warnakulasuriya S (2005). Oral submucous fibrosis: Review on aetiology and pathogenesis. Oral Oncol.

[B2] Sinor PN, Gupta PC, Murti PR, Bhonsle RB, Daftary DK, Mehta FS (1990). A case-control study of oral submucous fibrosis with special reference to the aetiologic role of areca nut. J Oral Pathol Med.

[B3] Lai DR, Chen HR, Lin LM, Huang YL, Tsai CC (1995). Clinical evaluation of different treatment methods for oral submucous fibrosis. A 10-year experience with 150 cases. J Oral Pathol Med.

[B4] Rajalalitha P, Vali S (2005). Molecular pathogenesis of oral submucous fibrosis-a collagen metabolic disorder. J Oral Pathol Med.

[B5] Mehrotra R, Pandya S, Chaudhary AK, Kumar M, Singh M (2008). Prevalence of oral premalignant and malignant lesions at a tertiary level hospital in Allahabad, India. Asian Pac J Cancer Prev.

[B6] Pindborg JJ, Odont, Sirsat SM (1966). Oral submucous fibrosis. Oral Surg, Oral Med, Oral Pathol.

[B7] Reichart PA, Philipsen HP (2006). Oral submucous fibrosis in a 31-year-old Indian women: first case report from Germany. Mund Kiefer Gesichtschir.

[B8] Kumar KK, Saraswathi TR, Ranganathan K, Devi MU, Elizabeth J (2007). Oral submucous fibrosis: A clinico-histopathological study in Chennai. Indian J Dent Res.

[B9] Hazarey VK, Erlewad DM, Mundhe KA, Ughade SN (2006). Oral submucous fibrosis: study of 1000 cases from Central India. J Oral Pathol Med.

[B10] Zhang X, Reichart PA (2007). A review of betel quid chewing, oral cancer and precancer in Mainland China. Oral Oncology.

[B11] Mehrotra R, Singh M, Kumar D, Pandey AN, Gupta RK, Sinha US (2003). Age specific incidence rate and pathological spectrum of oral cancer in Allahabad. Indian J Med Sci.

[B12] Babu S, Bhat RV, Kumar PU (1996). A comparative clinico-pathological study of oral submucous fibrosis in habitual chewers of pan masala and betelquid. J Toxicol Clin Toxicol.

[B13] Thomas G, Hashibe M, Jacob BJ, Ramadas K, Mathew B, Sankaranarayanan R (2003). Risk factors of multiple oral premaliganant lesions. Int J Cancer.

[B14] Ho PS, Yang YH, Shieh TY, Huang IY, Chen YK, Lin KN (2007). Consumption of areca quid, cigarettes, and alcohol related to the comorbidity of oral submucous fibrosis and oral cancer. Oral Surg Oral Med Oral Pathol Oral Radiol Endod.

[B15] Auluck A, Rosin MP, Zhang L (2008). Oral submucous fibrosis, a clinically benign but potentially malignant disease: report of 3 cases and review of the literature. J Can Dent Assoc.

[B16] Bhurgri Y (2005). Cancer of the oral cavity – trends in Karachi South. Asian Pac J Cancer Prev.

[B17] Reichart PA, Way TH (2006). Oral cancer and pre-cancer in Myanmar: a short review. J Oral Pathol Med.

[B18] Chiu CJ, Lee WC, Chiang CP, Hahen U, Kuo YS, Chen CJ (2002). A scoring system for the early detection of oral sub mucous fibrosis based on a self administered questionnaires. J Pub Health Dentist.

[B19] Maher R, Lee AJ, Warnakulasuriya KA, Lewis JA, Johnson NW (1994). Role of areca nut in the causation of Oral submucous fibrosis: A case control study in Pakistan. J Oral Pathol Med.

[B20] Gupta PC, Sinor PN, Bhonsle RB, Pawar VS, Mehta BC (1998). Oral submucous fibrosis in India: A new epidemic?. Natl Med J India.

[B21] Shah N, Sharma PP (1998). Role of chewing and smoking habits in the aetiology of oral submucous fibrosis (OSF): a case control study. J Oral Pathol Med.

[B22] Sur TK, Biswas TK, Ali L, Mukherjee B (2003). Anti-inflammatory and anti-platelet aggregation activity of human placental extract. Acta Pharmacol Sin.

[B23] Rajendran R, Rani V, Shaikh S (2006). Pentoxifylline therapy: a new adjunct in the treatment of oral submucous fibrosis. Indian J Dent Res.

[B24] Haque MF, Meghji S, Nazir R, Harris M (2001). Interferon gamma (IFN-gamma) may reverse oral submucous fibrosis. J Oral Pathol Med.

[B25] Kumar A, Bagewadi A, Keluskar V, Singh M (2007). Efficacy of lycopene in the management of oral submucous fibrosis. Oral Surg Oral Med Oral Pathol Oral Radiol Endod.

[B26] Nayak DR, Mahesh SG, Aggarwal D, Pavithran P, Pujary K, Pillai S (2008). Role of KTP-532 laser in management of oral submucous fibrosis. J Laryngol Otol.

